# Farmer Perceptions and Responses to Soil Degradation in Swaziland

**DOI:** 10.1002/ldr.2595

**Published:** 2016-09-13

**Authors:** Steven E. Orchard, Lindsay C. Stringer, Absalom M. Manyatsi

**Affiliations:** ^1^ Sustainability Research Institute (SRI), School of Earth and Environment University of Leeds Leeds LS2 9JT UK; ^2^ Agricultural and Biosystems Engineering Department, Faculty of Agriculture University of Swaziland Luyengo M205 Swaziland

**Keywords:** erosion, land degradation, natural resource management, semi‐arid, sustainable development

## Abstract

Soil degradation is globally concerning due to its adverse effects on the environment and agricultural production. Much of Swaziland is at risk from degradation. This paper assesses farmer perceptions and responses to soil degradation in 2002 and 2014, focusing on two land uses that underpin rural livelihoods: arable land and rangeland areas. It uses repeat household surveys and semi‐structured interviews, in two case study chiefdoms in the country's middleveld (KaBhudla and Engcayini) in the first longitudinal study of its kind. We find that observations of land degradation are perceived mainly through changes in land productivity, with chemical degradation occurring predominantly on arable land and physical degradation and erosion mainly in rangeland areas. Changes in rainfall are particularly important in determining responses. While perceptions of the causes and impacts of degradation largely concur with the scientific literature, responses were constrained by poor land availability, shorter and more unpredictable cropping seasons because of changing rains and low awareness, access to or knowledge of agricultural inputs. We suggest that sustainable arable land management can be enhanced through improved access to alternative sources of water, use of management practices that retain soil and moisture and greater access to agricultural inputs and capacity building to ensure their appropriate use. We suggest collaborative management for settlement planning that integrates soil conservation and livestock management strategies such as controlled stocking levels and rotational grazing could improve land quality in rangeland areas. Together, these approaches can help land users to better manage change. © 2016 The Authors. *Land Degradation & Development* published by John Wiley & Sons Ltd.

## Introduction

Land degradation can be defined as a persistent deterioration of land's productivity and often focuses on the soil component (Adeel *et al.,* 2005). Degradation is caused by multiple factors, both biophysical (e.g. climate, topography, hydrology and soil characteristics) and human (e.g. land use and management, policies and governance, migration, poverty and natural resource exploitation). These factors interact over multiple spatial and temporal scales (Kiage, 2013). Processes of land degradation have been observed across the globe, including in South America (Torres *et al.,* 2015), Asia (Xie *et al.,* 2015) and Africa (Stringer & Dougill, 2013). Reviews of global land degradation indicate that Africa is particularly vulnerable (Obalum *et al.,* 2012; Reed & Stringer, 2016).

Agro‐pastoralism is the predominant land use system in Africa (Hein, 2006). Rangelands cover approximately 30% and arable land approximately 8% of Africa's 30.2 million km^2^ land area (FAO, 2015). Over 75% of arable land in Africa is considered degraded (Khan *et al.,* 2014), while agricultural yields are relatively low because of soils characterised by poor fertility and overuse. Farming is also predominantly rainfed, so highly sensitive to changes and variability in the climate (Nyakudya & Stroosnijder, 2015). Reductions in agricultural productivity due to climate and other environmental changes could lead to yield deficiencies from rainfed agriculture of up to 50% during the period 2000–2020 (Li *et al.,* 2009), with related incomes dropping by up to 90% of 2002 levels by 2100 in some Southern African countries (Benhin, 2006). These dynamics and feedbacks between the climate and land degradation will have significant negative implications for rural livelihoods (Reed & Stringer, 2016) and will be felt largely at the local level by agro‐pastoralist households, for whom the land underpins their very survival (Pricope *et al.,* 2013).

Stringer (2014) presents a framework for analysing the structures and processes that can combine to shape different types of land and soil degradation at the local level. The framework highlights how present levels of land and soil degradation manifest locally are closely linked to past and present human (i.e. land management, local capacity and demographics) and biophysical (i.e. rainfall, topography and biota) aspects. Based on the framework, three types of soil degradation are distinguished and considered in this study: chemical degradation, physical degradation and erosion. Each can reduce ecological productivity and the economic benefits gained from land (Stringer, 2014). First, chemical degradation encompassing nutrient and organic matter losses can negatively influence soil structure, stability, water holding capacity, ecology and biodiversity. Second, physical degradation can take the form of soil compaction, sealing or alteration to the spatial distributions of soil biota. Finally, erosion is separated out as a subcategory of physical degradation because of its importance, particularly in the context of this research which focuses on Swaziland. Erosion can be attributed to both natural and human factors that result in pressure from wind and water removing fertile topsoil, particularly on sloping ground such as that found in the hills of the study area. Propensity for each type of degradation to occur locally depends on interactions between biophysical and human structures and processes operating across a range of scales (Stringer, 2014).

Soil degradation can be examined in many ways from the perspectives of several different stakeholders, including biophysical and social scientists, governments, non‐governmental organisations, development donors and land users (Warren, 2002). Whether soil changes are perceived as degradation or not depends largely on the management goals of the system of focus (Reed *et al.,* 2015). This suggests that degradation cannot be judged independently of its spatial, temporal, economic, environmental and cultural context (Sallu *et al.,* 2010). This paper focuses on the perspectives of land users themselves. It aims to assess farmer perceptions of and responses to degradation in 2002 and 2014, focusing on two key types of land use in Southern Africa: arable land and rangeland. We focus on Swaziland, taking a mixed methods approach, drawing on repeat household surveys and semi‐structured interviews, in two case study chiefdoms in the country's middleveld: KaBhudla and Engcayini. Our study offers significant novelty. This is the first study to take such a longitudinal approach using a mixed methodology to examine land user perspectives over a 12‐year time frame in Swaziland. We assess farmer perceptions of the causes of environmental changes and degradation on rangeland and arable areas in 2002 and 2014 and consider how farmers' responses impact upon land degradation processes. Extending knowledge in this area is both novel and vital in order to identify and understand the dominant factors that facilitate and inhibit agricultural production, sustainable livelihoods and sustainable land management practices. If we can understand those factors, it can help to guide policy development (Stringer & Dougill, 2013) so that rural households can move towards more sustainable land use and land management practices, as well as more resilient agricultural livelihoods, over the longer term (Sallu *et al.,* 2010).

## Materials and Methods

### Study Country: Swaziland

Swaziland has a population of 1,419,623 and total land area of 17,364 km^2^ (CIA, 2015), of which over half is at risk from degradation (World Bank, 2015a). Seventy‐one percent of this land is under agricultural use, characterised by arable crop farming and livestock production (FAO, 2013). Two principal types of land tenure dominate Swazi Nation Land (SNL), held in trust for the nation by the King, accounts for 56% of the land area (Mavimbela *et al.,* 2010). SNL for small‐scale arable agriculture is allocated by the chief to household heads, traditionally a married male (Funnell, 1991). The proportion of land received depends on a person's needs, age, social status and lineage. The majority of the population (approximately 75%) engages in subsistence agriculture on SNL (Xaba & Masuku, 2013), where they produce crops for household consumption, selling surpluses. The principal food crop is maize (*Zea mays*) but others (e.g. groundnuts, dry beans, sorghum, pumpkins, jugo beans, soya beans and sweet potatoes) are also grown (Mavimbela *et al.,* 2010). Title Deed Land (TDL) is distinguished by recognition of exclusive access rights to defined areas, with titles held by either corporate bodies or individuals (Mushala *et al.,* 1998), and accounts for 44% of the land area (Mavimbela *et al.,* 2010). TDL is used to grow high value commercial crops (e.g. sugar, trees for timber and paper production and citrus fruit). TDL is characterised by high levels of investment and irrigation, and high productivity. This paper focuses on SNL.

Sixty‐three percent of Swaziland's population lives below the average poverty line ($2 per day), with approximately 29% below the extreme poverty line ($1.25 per day) (World Bank, 2015b). Declining soil fertility has contributed to decreasing agricultural productivity at the national scale (Mashinini *et al.,* 2011). Although hybrid maize occupies >80% of cropped SNL (Naysmith *et al.,* 2009), its impact on national food production has been negligible: <5% is irrigated and fertiliser use is low (Terry, 2012). Poor productivity is also exacerbated by erratic rainfall patterns and heatwaves impacting crop development, lack of diversification, invasive weed species, fires, weak markets and monopolies that increase agricultural input prices, a lack of appropriate research, HIV and AIDS that reduce labour and poor land use planning (Mavimbela *et al.,* 2010). Approximately 81% of the country's total cattle population is owned by SNL farmers. In Swazi culture, livestock enhance social status, being viewed as a store and measure of wealth and are used as a medium of exchange in marriages. Poorer people cannot afford cattle (Stringer *et al.,* 2007a). Livestock manure derived from night kraaling, whereby livestock are herded into pens overnight to protect them from predators and theft, is a valuable source of organic fertiliser (Stringer *et al*., 2007a).

### Research Design

Data were collected during May‐October 2002 and September‐October 2014, in two chiefdoms: Engcayini and KaBhudla. Both are located in Swaziland's middleveld, where nearly half the population lives on a quarter of the country's land area (Hutcheson, 2008). The middleveld has been repeatedly identified as experiencing severe degradation (Stringer, 2009).

Engcayini comprises land with ‘good to fair’ production potential (Mushala *et al.,* 1998), while soils comprise Rhodic Ferralsols and Saprolites with patches of acid clay (Jansen *et al.,* 1994). KaBhudla is at lower altitude than Engcayini, and soils have variable clay content linked to heterogeneity within the underlying metamorphic bedrock (GOS, 1993). Across the two study areas, arable plots and communal rangeland are the dominant land uses. Households typically cultivate maize, complemented with groundnuts (*Arachis hypogaea*), beans (*Vignia* spp.) and/or sweet potatoes (*Ipomoea batatas*) (Stringer *et al.,* 2007a, 2007b). Livestock owners use communal areas for grazing, while poorer families use rangelands to collect wild grasses, herbs and fruit, which they sell to gain additional income (Stringer *et al*., 2007a, 2007b). Following the maize harvest and an announcement by the chief, distinction between the land uses becomes blurred. Maize stalks are left in the arable plots, and cattle are allowed to graze them, so all land effectively becomes open access. This links the two different land uses and allows cattle to naturally return nutrients to the soil through their manure. This practice is facilitated by a lack of fences on arable plots (Stringer, 2004).

KaBhudla was under the leadership of Chief Magutjwa Magagula in 2002, but he passed away in 2008. A new chief was yet to be appointed at the time of data collection in 2014 (personal communications with village leaders). Engcayini has remained under leadership of a Prince (Royal Dlamini family) during both periods of data collection. Social, political, administrative and institutional changes are slow moving in Swaziland and remain the same during the study period. Traditions and cultural factors remain strong in informing social practices, particularly in Engcayini, while extension advice in the study areas remained weak. Roads and transport infrastructure were poor in Engcayini in both 2002 and 2014 but were slightly improved in KaBhudla by 2014. Market access remained comparable over the study period in both study areas.

Household questionnaire surveys were used to gather data from a large number of respondents (see Stringer, 2004 for complete list of questions, as well as Table 1 in the results section). Surveys were conducted with household heads (2002 *n* = 174 and 2014 *n* = 184) to identify livelihood strategies and land use patterns during each period of data collection. Heads were those individuals in charge of managing the household at the time of data collection. In Engcayini, all households were surveyed in both 2002 (*n* = 74) and 2014 (*n* = 84). In KaBhudla, a sample of households (*n* = 100) out of a total of 121 households (GOS, 2007) was surveyed both times. Sample sizes aimed to capture the diversity within each community while being both representative and achievable given available time and resources. In KaBhudla, households were selected randomly but stratified proportionately according to accessibility from the main road. As most homesteads were located along the main gravel roads, proportionally more households were interviewed along the roads than in the remote areas. The same (largely closed) questions were used during both periods of data collection to obtain information on livelihoods, land use practices and environmental changes and priorities. Data were converted into percentages, and descriptive statistical summaries were developed. These were then used to calculate percentage changes in perceptions using Microsoft Excel 2010 and were compared with reveal similarities and differences between 2002 and 2014.

**Table 1:** Illustrative quantitative data regarding land degradation

	Engcayini			KaBhudla			Overall		
	2002 (*n* = 74) (%)	2014 (*n* = 84) (%)	Change (%)	2002 (*n* = 100) (%)	2014 (*n* = 100) (%)	Change (%)	2002 (*n* = 174) (%)	2014 (*n* = 184) (%)	Change (%)
1. Did you grow maize last year?									
Yes	100	100	0	82	75	−7	90	86	−6
No	0	0	0	18	25	+7	10	14	+4
2. Was it enough land to provide food for your family last year?									
Yes	49	75	+26	51	12	−39	50	40	−10
No	51	25	−26	49	88	+39	50	60	+10
3. Has your maize yield increased in the last 5 years?									
Yes	22	11	−11	0	0	0	9	5	−4
No	58	38	−20	94	66	−28	79	45	−34
Varies	19	43	+24	3	23	+20	10	29	+19
4. Why do you think your crops failed?									
Drought	–	35	**–**	–	88	**–**	–	59	**–**
Lack of inputs	–	68	**–**	–	6	**–**	–	26	**–**
Other	–	8	**–**	–	7	**–**	–	7	**–**
5. Do you apply manure?									
Yes	66	64	−2	56	56	0	60	53	−7
No	34	36	+2	44	44	0	40	47	+7
6. Do you keep cattle?									
Yes	68	55	−13	33	41	+8	48	47	−1
No	32	45	+13	47	59	−8	52	53	+1
7. What are the reasons you keep livestock?									
Tradition	10	24	+14	9	63	+54	6	20	+14
Bank	14	4	−10	42	34	−12	14	9	−5
Food	73	30	−43	58	37	−21	41	16	−25
Manure	10	61	+51	3	44	+41	5	25	+20%
Draught	42	50	+8	24	0	−24	22	13	−9
8. How have cattle numbers changed in last 5 years?									
Increased	28	39	+11	27	41	+14	13	15	+2
Decreased	72	61	−11	61	59	−2	31	22	−9
9. If numbers decreased, why?									
Died	97	72	−25	85	19	−66	28	11	−17
Sold	1	20	+19	10	81	+71	2	10	+8
Stolen	–	4	**–**	–	6	**–**	–	2	**–**
Lobola	–	8	**–**	–	6	**–**	–	2	**–**
Other	–	0	**–**	–	0	**–**	–	0	**–**
10. What other crops did you grow?									
Groundnuts	13	42	+29	2	18	+16	4	29	+25
Sweet potatoes	18	20	+2	2	31	+29	5	26	21
Beans	2	11	+9	4	9	+5	3	10	+7
11. What do you use to plough?									
Tractor	81	82	+1	93	98	+5	80	85	+5
Ox	9	6	−3	6	0	−6	7	3	−4
Tractor and ox	9	12	+3	1	0	−1	5	5	0
Other	–	0	0	–	1	**–**	–	1	**–**
12. Do you apply manure?									
Yes	66	64	−2	56	56	0	60	53	−7
No	34	36	+2	44	44	0	40	47	+7
13. Do you apply fertilisers?									
Yes	85	65	−20	55	52	−3	68	52	−16
No	15	35	+20	45	48	+3	32	48	16
14. Do you water your crops?									
Yes	0	4	+4	0	2	+2	0	3	+3
No	100	96	−4	100	98	−2	100	97	−3
15. Do you use a tractor to plough?									
Yes	81	82	+1	93	98	+5	80	85	+5
No	19	18	−1	7	2	−5	20	15	5
16. Is soil erosion a problem for you?									
Yes	–	84	**–**	–	96	**–**	–	98	**–**
No	–	16	**–**	–	4	**–**	–	2	**–**
17. Have you ever seen the soil being washed away by rain?									
Yes	74	96	+22	76	96	+20	75	96	+21
No	26	4	−22	24	4	−20	25	4	−21
18. What causes soil erosion?									
Rain	59	71	+12	76	97	+21	69	85	+16
Cattle tracks	42	46	+4	9	2	−7	23	22	−1
Slopes	14	7	−7	–	0	–	–	3	–
Poor road drainage	3	4	+1	–	3	–	–	2	–
Bare ground	–	26	**–**	17	0	−17	–	12	–
Ploughing	–	0	**–**	–	0	–	–	0	–
Do not know	1	1	0	5	0	−5	3	1	−2
Other	–	2	**–**	22	0	−22	–	1	–
19. Do you carry out any actions to reduce soil erosion?									
Yes	96	42	−54	83	46	−37	89	44	−45
No	4	58	+54	17	54	+37	11	56	+45
20. Are grass strips good at stopping erosion?									
Yes	76	100	+24	–	92	**–**	–	96	**–**
No	8	0	−8	–	4	**–**	–	2	**–**
Do not know	16	0	−16	–	4	**–**	–	2	**–**

Additionally, transect walks, seasonal calendars and semi‐structured interviews were conducted with three case study households in each chiefdom (total *n* = 6 each time). Five of the same case study households participated in 2014 as in 2002, at which time, their involvement in the research was based on them having access to up to 4 ha of land and having been established in the area over 20 years. Each household represented a different level of wealth and income relative to other households in the chiefdom (Stringer, 2004). In KaBhudla, two households along the main road were selected and one in a more remote part. By 2014, one case study household had relocated away from KaBhudla. Based on preliminary analysis of the household questionnaire data from 2014, another household with comparable characteristics was sampled to maintain the coherence of the longitudinal approach.

Transect walks through arable plots with case study household heads aided familiarisation with local context, history and issues relating to each community and helped to organise and refine spatial information. Seasonal calendars provided a temporal dimension to resource use and land management decisions (Woodward *et al.,* 2012). Both seasonal calendars and transect walks also enabled more targeted questioning for subsequent semi‐structured interviews. Semi‐structured interviews with each case study household provided in‐depth past and present perspectives on degradation and its impacts during the two snapshots in time (2002 and 2014). Semi‐structured interviews elucidated the following: (i) household land use and land management practices; (ii) how degradation is recognised and has affected livelihood decisions and responses; and (iii) how aspects identified in (i) and (ii) interact with local level political, socio‐economic and environmental aspects. Qualitative data were analysed by first indexing the data under categories of the framework presented by Stringer (2014). Patterns within themes were then identified, with similar ones grouped together. While not explicitly contributing to theory development, this approach borrowed from the qualitative content analysis aspects of grounded theory (Corbin and Strauss, 1990), as categories emerged through iterative data analysis, refining the codes as new data were evaluated. Results were then tabulated. Quantitative and qualitative data were compared and contrasted in order to elucidate an overall narrative of change, which is presented in our Results section and structured according to Stringer's (2014) framework, which underpins the research.

Sampling biases may lead to a distortion in the results obtained during the process of data collection (Varkevisser *et al.,* 2003). Occasionally, farmers' perceptions of environmental change are inconsistent with physical data (Simelton *et al.,* 2013). Farmers tend to recall changes as being more recent than physical data indicate. This is illustrated by Marx *et al.* (2007), who show that while memories of extreme weather events are vivid if they coincide with other memorable events (the availability heuristic), decisions are typically based on recently experienced events and hence over estimate the likelihood of the same event reoccurring (the recency heuristic). Perceptions can be confounded by factors such as failure to differentiate between changes in the exposure to and impacts of environmental change and the sensitivity of the farming system to environmental change (Simelton *et al.,* 2013). To address these challenges, a balanced reflection on the problems of using perceptions was sought, and data were triangulated. Iterative reflections were carried out jointly with case study households through focus groups in each study chiefdom in June 2015, determining how and why any conflicts in information may have occurred and validating the findings.

### Results

In this section, we use Table 1 to present the quantitative data and Table 2 to present illustrative qualitative data. The accompanying narrative is structured according to the themes which emerged through the integrated analysis. In both 2002 and 2014, households primarily perceived land degradation through arable and rangeland productivity. Results from the household questionnaire survey (Table 1) show that in Engcayini, 50% of households perceived that they produced enough food for household consumption in 2002, increasing to 75% in 2014 (question 2, Table 1). Increased variability in annual yields was mainly attributed to changes in the use of agricultural inputs. Conversely, in KaBhudla, 50% of households perceived that they produced enough food in 2002, falling to approximately 10% in 2014. Increased variability of annual yields was largely linked to changing rainfall patterns during interviews (Table 2).

**Table 2:** Illustrative qualitative data regarding land degradation

	Engcayini household quotes	KaBhudla household quotes
Arable land	‘We used to plough with the first rains then plant with the second rains. But the rain is so unpredictable now it is too risky to do this. We have to plough and plant with the first rain, which is not good for the crops’. (HH1E) ‘The rain often falls in short heavy bursts now, which doesn't sink into the soil to allow us to plough and washes the topsoil away causing much damage’. (HH3E) ‘There is less for the cows to feed on now and they are becoming thin and weak, and the males are even infertile. They catch diseases easily but we can't afford the vaccines’. (FGE) ‘Weed and pest infestations are getting worse. They attack our crops, and we remove them by hand but they always come back. It's like fighting a losing battle’. (HH2E) ‘Even if you have money for inputs, we don't know what to buy or how to use them. In the past extension officers lived in the communities and when we had problems we went to the extension officer's house to get advice. But now they are far away in town, and if you wait for the extension officer to come from town to give you advice you might miss the first rains’. (HH2E) ‘We use foreign seeds now. They can survive much better than Swazi seeds. But they need much more maintenance which is expensive, often more than you get from the harvest’. (HH3E)	‘Some households are able to get water from boreholes or store river water in tanks, but not everyone can do this. Even so, borehole water can be too salty for crops, and stored water is no good if it has been still for too long’. (HH1K) ‘If they were to build catchment areas that could be supported by piping, it could help people to have water towards their fields. If there can be a reservoir somewhere and we can increase the accessibility of people to water, that would be useful, with taps in their own homesteads. People could buy pipes to take water to their fields. More sand dams could help as well as they are not expensive’. (FGK) ‘Some people store fodder for the winter months, or use arable fields as private grazing pens, but these options are not available to everyone’. (HH3K) ‘Waiting for tractors is hard. They go to homesteads closer to the tractor hire place first, and often don't come here at all! You can wait two or three seasons without a tractor, and even if you have paid you don't get your money back. They say “next season, next season”. If they do pitch up, they come when it's dry and you can't do anything with it! Then you end up ploughing dry land because it's “now or never” ’. (FGK) ‘Government is driving diversification of crops. But it's hard to change the way we do things. Some farmers do change but very few. We always think it will be different next time and we won't have the same problems… farmers rely on food for survival, so if you don't cultivate maize you are missing your staple food’. (HH1K)
Rangeland	‘Farmers make tracks through fields when they take short cuts to get their cattle to the dip tank, that's when the gullies start. Overgrazing and heavy rains then make the gullies bigger, especially on steep hills. The gullies block roads and paths, make less grazing land, and then they come into our fields affecting our crops’. (HH2E) ‘The grass strips have always been there. Some people destroy them to make more space to plant, but they regret it when the soil erodes. There is a law enforcing grass strips, but not many people know about it and there is no punishment…it is up to the farmer if they want them or not’. (HH1E)	‘The urban–rural migration is making the dongas worse as people are even building next to the dongas. There used to be a land use policy but it's not that much effective. Homesteads are now built on grazing land and arable lands too. The policy should be stricter’. (FGK) ‘There is an invasive tree that has appeared in the past 10 to 15 years. It has spread very quickly. It takes all the nutrients from the soil so nothing grows around it, leaving nothing for cattle to graze on’. (HH2K)

### Rainfall, Topography and Biota

Biophysical differences in productivity between chiefdoms were largely due to differences in rainfall, topography and biota. Changing rainfall patterns were perceived as the most notable agricultural challenge overall in both time periods, namely, (1) increasing instance of late first rains (from arriving typically in July/August up to 60 years ago, to increased instance of occurrence of arriving as late as October/November between 2002 and 2014); (2) growing unpredictability of subsequent rains; (3) increased occurrence of drought periods; and (4) increased occurrence of intense rainfall. Households from both chiefdoms said they would benefit from access to alternative sources of water (rainfall storage, boreholes and water from nearby dams) to irrigate crops. These options are only available to a limited number of wealthier households at present. However, respondents noted that water stored from alternative sources can become stagnant, and borehole water is often too saline for irrigation, while significant alterations in rainfall patterns mean alternative sources of water are increasingly scarce (Table 2).

Soil erosion was a serious problem for a majority of households in both chiefdoms and time periods. Intense rainfall was perceived to be the greater driver of erosion in 2014. Increased periods of intense rainfall meant water did not soak into the soil, and a greater percentage of households had also seen soils washed away by rain in 2014 compared with 2002 (Table 1), taking the topsoil and its nutrients with it. Households from both chiefdoms perceived erosion on arable land caused by intense rainfall to be facilitated by inadequate roadside drainage causing runoff, periods of drought followed by periods of heavy rainfall and encroachment of gullies into arable areas from neighbouring communal grazing land. Rangeland gullying was perceived as more problematic in both chiefdoms in 2014 than in 2002. Respondents explained that reduced vegetation due to drought contributed to topsoil losses because of intense rainfall in both chiefdoms and time periods, resulting less grazing for cattle (Table 2). A severe cyclone in 1984 was highlighted in both chiefdoms during both periods of data collection as having accelerated the expansion of existing gullies and their encroachment into arable fields. Engcayini's steep hill slopes facilitated erosion.

Households noted greater prevalence of weeds such as *Striga asiatica* on arable land in 2014. Changes in rainfall patterns were recognised to contribute to increased *S. asiatica* infestations. On rangelands, invasive tree species were noted to spread quickly on degraded areas, reducing grass growth and leaving soils bare.

### Land Management

While historically (up to 60 years ago), households could plan their activities and spread ploughing, sowing, weeding and harvesting over the cropping season, changing rainfall patterns have significantly reduced the temporal window of these tasks. As shown in Table 2, ploughing, planting, weeding and harvesting times have been altered in light of changing rainfall patterns. Ploughing and sowing are now conducted simultaneously with the first rains rather than being spread over the first and second rains. Respondents reported during interviews that these practices increase pressure on soil, giving it less time to recover, and can increase future risks of crop failure. Crop rotation, intercropping, fallow periods and crop diversification were practised to varying degrees in both chiefdoms and time periods to improve soil fertility, with greater groundnut cultivation in 2014 in both locations. While farmers recognised the benefits of such activities, reduced land availability and high perceived risk of crop failure significantly constrained their practice. Greater crop diversification was observed in KaBhudla as a risk‐spreading strategy because of lower rainfall available for growing maize.

Slightly more households applied manure in Engcayini (approximately 66%) compared with KaBhudla, in both time periods (Table 1). While there was a decline in households keeping cattle in Engcayini compared with a rise in KaBhudla, there was a perceived decrease in total cattle numbers in both chiefdoms. Decreases were perceived to result predominately from die‐off in 2002, compared with the sale of cattle in 2014. Although cattle are not traditionally sold, increased agricultural uncertainty has increased animal sales, raising funds for people to buy fodder, hire tractors and pay school fees and medical bills. Furthermore, the main reasons for keeping cattle in both chiefdoms changed from a source of food in 2002 to a source of manure in 2014. Manure was increasingly scarce in both chiefdoms because of perceived declines in cattle numbers, which also links to reduced rangeland quality if cattle are thin and weak (Table 2). This has caused cattle to become more susceptible to disease and resulted in fewer cattle for many households. Although vaccines may be available for certain diseases, farmers have little awareness about them. Vaccines are often expensive, leaving weak cattle even more vulnerable if they are not routinely vaccinated. Even during mating season (October), bulls return hungry and unfit to mate, further contributing to lower cattle numbers.

Reductions in manure availability meant that it is typically only applied to fields close to homesteads. However, interviewees reported that these fields experienced higher infestations of cutworm (*Busseola fusca*), which feeds on organic matter and attacks the stems of seedlings. Fields further from homesteads typically receive less manure and organic matter and are less fertile but experience fewer issues with *B. fusca.* Nevertheless, more distant fields face greater challenges with *S. asiatica*. Respondents stated that *S. asiatica* reduces crop yields by taking water and nutrients away from crops.

### Local Capacity

While qualitative data suggested that farmers perceived inorganic fertilisers to reduce soil quality and create dependency on their use in both chiefdoms, quantitative data indicated that most households applied inorganic fertiliser in Engcayini in both data collection periods (although falling from 85% to 65%), with approximately 50% doing so in KaBhudla in both years (Table 1). Households identified lime, seed and fertiliser availability; affordability; and knowledge of appropriate use as significant concerns in both chiefdoms and time periods. To increase yields from less fertile soils, some farmers have switched to different seed varieties, which respondents stated are more resilient to drought, weeds and pest; mature quicker and are less sensitive to rainfall fluctuations; can grow in lower quality soils; and the maize produced can be stored for longer. Farmers often said they select seed varieties that they see working well for surrounding farms or hear advertised on the radio.

In Engcayini in 2002, some farmers explained how they joined a cooperative to receive inputs to improve soil quality and yields. Despite positive outcomes, high loan repayment rates meant that many farmers had left the cooperative by 2014. A majority of farmers stated that they do not receive any government extension advice so even if they had the money they would not know which inputs (e.g. seeds and fertiliser) to purchase for the best possible yields. They either bought inputs that had been successful for other farmers or purchased whatever was locally available. Furthermore, farmers often lacked the necessary knowledge to apply inputs correctly, mixing fertiliser and manure arbitrarily or retaining seeds from previous harvests to save money.

Although erosion is considered serious, reduced numbers of households carry out actions to combat it, declining from a majority of households in 2002 to less than half in 2014 in both chiefdoms. Rangeland gullies are typically ignored until they cut across roads or paths, or encroach into arable fields. While there have been instances of collective action to rehabilitate gullied areas, without state or non‐governmental organisation support in both chiefdoms, these groups typically break down. Extension advice on how to tackle soil erosion was greatly lacking. Households in both chiefdoms typically used trial and error or methods that have worked for nearby households. Responses included planting trees and aloes to stabilise soil, digging furrows to divert rain water away from fields and creating grass strips to retain the soil when it washes downslope.

A majority of households in both chiefdoms considered grass strips effective in combating erosion. In Engcayini, grass strips also provide grass for thatching and mat‐making, whereas in KaBhudla, they provide fodder and demarcate fields. Households in Engcayini stated that the grass strips on their land had been there since they first settled in the area, with one household stating that they were informed of the benefit of grass strips by extension officers who told them where to place them and how wide to make them. This suggests that in the past, extension advice was received. In both chiefdoms, disadvantages of grass strips were identified, such as reducing the area of arable land; making ploughing more difficult by breaking up the land into smaller parcels; and attracting pests and weeds. Households typically burned grass strips before arable fields were ploughed to kill any pests and weeds they contained.

### Demographic Change

Households in both chiefdoms and time periods identified increased settlement as a driver of degradation. Rural in‐migration (i.e. those leaving urban areas to establish homesteads in rural areas) was of particular concern in KaBhudla in 2014 because of its closer proximity to Manzini (the nearest city) and its significantly lower level of municipal rates. Respondents explained that in Swazi culture, everyone is entitled to enough land to provide a home and to grow food for the family. After the chief agreed to an allocation (usually taken from communal grazing areas), many migrants requested additional land to cultivate food, further reducing the rangeland.

Rangeland gullying was more problematic in both chiefdoms in 2014 than in 2002. While cattle numbers are perceived to be lower in 2014 than in 2002, overgrazing was still seen as problematic. This is predominantly due to smaller rangeland areas as a result of increased settlement, and reduced vegetation due to drought. Greater concentration of livestock has caused greater prevalence of cattle tracks, particularly as the animals are herded to dip tanks. Trampling removes vegetation, leaving land bare and exposed to rainfall that washes away the soil. Many households noted that cattle tracks are a precursor to gullies, but that they continue to use the shortest and quickest routes to the dip tank. Overgrazing also increased on the reduced rangeland area, resulting in gullies that are then accelerated by heavy rain. Households noted that cattle sometimes fall into gullies while straying too close to the edge in search of food. Most households stated that fences were required to contain cattle and that rotational grazing strategies would help combat overgrazing. However, attempts to introduce this in Engcayini in the early 2000s had been unsuccessful. Deforestation was another major concern in both chiefdoms, with growing populations resulting in more demand for firewood, increasing pressure on forests and exposing soil to erosion.

Solutions to reduce gullying suggested by research participants include settling new homesteads into the same area so not to fragment grazing land. Settlement decisions are made by the chief and his advisers. Some respondents argued that the community should be involved in decision making too as the consequences impact their livelihoods and ability to support their families.

## Discussion

### Chemical Degradation

Observations of chemical degradation were greater in 2014, occurred predominantly on arable land and were of greatest concern to farmers. Farmer perceptions of increasingly unpredictable rainfall are comparable with evidence of high variation in spatial and temporal patterns of both total annual and planting season rainfall in Swaziland (Oseni & Masarirambi, 2011). Increased risk of crop failure that farmers attributed to changes in rainfall, in the form of poor seed germination, washing away of seeds and crops, stunted growth and drying of crops, has been documented by Lema & Majule (2009) in their study of the impacts of climate variation and change on agriculture in semi‐arid areas of Tanzania, suggesting that our findings are not specific to our study sites. Such issues will be exacerbated with climate change projections indicating reduced cultivation times of >20% by 2050 compared with 2006 (Thornton *et al.,* 2014). Furthermore, local observations of increasing *S. asiatica* infestations also link to rainfall, particularly as the weed is drought tolerant, growing well in areas receiving <1,500 mm rainfall (Stringer *et al.,* 2007b).

Farmers expressed a link between intensive land management practices and depletion of soil nutrient and organic matter content (Vanlauwe *et al.,* 2014). In line with research from South Africa (Zingore *et al.,* 2011), farmers stated that soil fertility issues result from continuous cultivation and soil nutrient removal. Farmers explained that soil quality decline could be tackled with low input practices, such as improved fallows, using legumes in rotation or intercropping to restore soil nutrients (cf. Assefa & Hans‐Rudolf, 2015). However, the perceived high risks of crop failure due to unpredictable rains and reduced land availability have been shown to severely restrict these options (FAO/WFP, 2005). Furthermore, lack of timely and affordable access to and knowledge of agricultural inputs constrained many farmers (cf. Giger *et al.,* 2015). Barrett (2008) illustrates how soil quality risks a downward spiral of degradation, as reductions in agricultural yields due to rainfall alteration can undermine households' abilities to invest in inputs, causing further declines in soil quality. Sanchez (2010) states that efforts to replenish soil fertility are a primary requirement for breaking the cycle of poverty and increasing food security in Africa.

### Physical Degradation

Observations of physical degradation were greater in 2014 and occurred predominantly on rangelands. Physical degradation was perceived to be largely the result of increased settlements, livestock trampling and invasive tree species. While research from elsewhere highlights a positive relationship between soil quality decline and rural out‐migration (Gray, 2011), in our study chiefdoms, building and construction resulting from rural in‐migration caused physical degradation through soil sealing. Beauchemin (2011) suggests rural in‐migration is linked to recessive economies and increased socio‐economic hardship for urban residents. However, our results demonstrate the ‘pull factor’ of lower land tax rates compared with nearby municipalities. Subsequent sealing of soils due to impervious settlement construction contributes to climate change by affecting energy transfers, temperature regulation and the propensity of soil to act as a carbon sink (Stringer, 2014). Settlements had also fragmented and reduced the available rangeland areas, causing cattle densities to increase. While recent research from South Africa suggests that greater population densities correlate with higher levels of vegetation and reduced erosion (Kiage, 2013), our results show the opposite (cf. Hobbs *et al.,* 2008). Subsequent soil compaction through trampling and the creation of tracks had disrupted water flows (cf. Swanepoel *et al.,* 2015). Indeed, soil sealing and compaction have been shown to impact soil porosity, either reducing or modifying it, with knock‐on effects on wider ecosystem functions and processes (Scalenghe & Marsan, 2009), which farmers observed through reduced rangeland productivity. Drought and overgrazing together contributed to the increase of invasive tree species on communal rangeland and corresponds with research highlighting links between overgrazing and bush encroachment in nearby Botswana (Reed *et al.,* 2015). The spatial distribution of soil biota has been shown to shape important soil functions, including water and nutrient cycling and the provision of biodiversity and habitat (Thomas & Dougill, 2006). Such findings are highly relevant to the Aichi Biodiversity Targets under the Convention on Biological Diversity as well as the Sustainable Development Goals (United Nations, 2015), which express the critical importance of biodiversity for supporting local livelihoods, reducing poverty and improving food security.

### Erosion

Observations of erosion were greater in 2014 and occurred predominantly on rangelands. Farmer perceptions of the causes of erosion are well supported in the literature (e.g. Lal, 2001; Teshome *et al.,* 2014). Hein (2006) notes that differences in such biophysical conditions, which shape the sensitivity of land to rainfall variability and change, present a significant challenge for estimating the impact of land management on erosion. In Swaziland, the King's Order of 1953 requires farmers to leave grass filter strips on all ploughed land. These must be approximately 2 m wide, at intervals of 5–20 m, depending on the gradient (Osunade & Reij, 1996). These represent a low‐cost measure that can help to reduce erosion in both arable and rangeland areas.

Existing research clearly describes link between cattle tracks and the formation of gullies (le Roux & Sumner, 2012). Such observations are comparable with findings from Mohammad & Adam (2010) in their study of the impact of vegetative cover type on runoff and soil erosion under different land uses. Deforestation in our study chiefdoms due to rising demand for fuel wood further contributed to erosion through vegetation loss and topsoil exposure, supporting findings from Kebede *et al.* (2010) in their study of energy consumption and economic development in sub‐Saharan Africa. Overgrazing and deforestation left space open for invasive plants to grow. While research highlights ecosystem service losses resulting from invasive tree species (Shackleton & Gambiza, 2008; Reed *et al.,* 2015), invasive shrubs on the rangelands in our study have reduced vegetation around tree trunks, further exposing topsoil and accelerating erosion (cf. Pannell & Vanclay, 2011). The prevailing socio‐economic context, particularly challenges of migration and poverty, contribute to erosion as well as physical degradation through soil sealing, as growing numbers rely on natural resources for their livelihoods, placing increasing pressure on reduced rangeland areas (Wagner *et al.,* 2015).

### Recommendations

A more comprehensive and appropriate set of criteria for identifying and understanding the causes and effects of different types of land degradation need to be developed, recognising how land use and land user capacities can shape land degradation and land management practices. Support should be provided for communities to utilise alternative sources of water such as boreholes, wells, springs and dams. As various institutional and organisational challenges can arise when managing water as a common good, efforts should be made to raise farmer capacity to implement sustainable land management practices that retain soil moisture levels, such as vegetative strips, terraces, ditches, water harvesting, zero/minimum tillage and mulching. These practices will help tackle chemical degradation and combat erosion on arable fields. Affordable and timely access to agricultural inputs should be provided to sustain farmer cooperatives, with locally available extension regarding the correct type, amount, use and application. Rural in‐migration should be properly regulated and controlled to reduce physical degradation and erosion on rangeland. Settlement planning should also integrate soil conservation measures, ensuring proper drainage of run‐off from impermeable road surfaces. Efforts should also be made to combat overgrazing on rangelands through the implementation of appropriate stocking levels and rotational grazing. Participation of farmers from the design stage of such initiatives is crucial to ensure that they have community buy‐in and are locally appropriate. Development agencies should ensure participation is collaborative in order to harness the knowledge of various stakeholders while not alienating or threatening the position of traditional leaders. Further research is required to understand the broader scale institutional structures and processes of land governance which shape land degradation, its management and its impact on rural livelihoods.

## Conclusions

Swaziland's population faces intractable challenges from land degradation. Farmers perceive that land degradation is occurring mainly through changes in land productivity. This in turn affects their livelihoods and their ability to feed their families. Farmers' perceptions suggest that chemical degradation occurs predominantly on arable land, while physical degradation and erosion have worsened over the study period and are concentrated mainly in rangeland areas. Each of these types of degradation has had impacts on livelihoods. Changes in rainfall were found to play key role in shaping farmers' responses to degradation. While perceptions of the causes and impacts of degradation broadly agree with those apparent in the scientific literature, farmers' responses were constrained by factors including poor land availability, shorter and more unpredictable cropping seasons because of changing rains and low awareness, access to or knowledge of agricultural inputs. The results we have presented in this paper highlight the urgent need for more sustainable land use and management planning, and our recommendations outline important ways forward.
